# Differences in resistance to 5-fluorouracil as a function of cell cycle delay and not apoptosis.

**DOI:** 10.1038/bjc.1995.519

**Published:** 1995-12

**Authors:** M. Pickard, C. Dive, A. R. Kinsella

**Affiliations:** Department of Surgery, University of Liverpool, UK.

## Abstract

**Images:**


British Journal of Cancer (1995) 72, 1389-1396
© 1995 Stockton Press All rights reserved 0007-0920/95 $12.00

### Differences in resistance to 5-fluorouracil as a function of cell cycle delay and not apoptosis

M Pickard[1], C Dive[2] and AR Kinsella[1]

[1]*Cellular Oncology Group, Department of Surgery, University of Liverpool, PO Box 147, Liverpool L69 3BX, UK;* [2]*Department of Physiological Sciences, University of Manchester, Stopford Building, Oxford Road, Manchester M13 9PL, UK.*

**Summary** A series of human embryo fibroblasts has previously been shown to display increasing resistance to the antimetabolites methotrexate (MTX) and *N*-phosphonacetyl-L-aspartate (PALA) with increasing tumorigenicity. This increased resistance was found to be further increased as a result of salvage pathway activity for purine and pyrimidine biosynthesis. A similar pattern of increasing resistance paralleling increasing tumorigenicity has now been shown to occur with 5-fluorouracil (5-FU), which is independent of salvage pathway activity. The KMS normal cell line was found to be more sensitive to 5-FU than either the immortalised KMST or tumorigenic KN-NM cell lines. Immunohistochemical analysis of the three cell lines demonstrated high levels of p53 protein in the KMST and KN-NM cell lines, but undetectable p53 levels in the KMS cell line. From these data it was hypothesised that a difference in p53 function may be causing the difference in the patterns of sensitivity observed in the three cell lines. P53 is now believed to function as a regulator of a $G_1$ to S cell cycle checkpoint and as an inducer of apoptosis following DNA damage to the cell. The differences in sensitivity of the cell lines could not be explained by differences in the levels of apoptosis but could be attributed to differences in cell cycle response. Our evidence suggests that loss of cell cycle control, possibly through loss of p53 function, is an important factor in increasing the drug resistance of fibroblast cell lines.

**Keywords:** drug resistance; 5-fluorouracil; p53; $G_1$ arrest; apoptosis

The resistance of tumour cells to chemotherapeutic agents is a major clinical problem in the treatment of human malignancy. Many mechanisms of resistance have been postulated and studied (Fox *et al.*, 1991). In the past, resistance of tumour cells to certain members of the antimetabolite group of drugs has been attributed to either gene amplification (Schimke *et al.*, 1977; Wahl *et al.*, 1979) or the increased activity of the salvage pathways for purine and pyrimidine biosynthesis (Weber 1983; Kinsella and Haran, 1991).

In fact previous work from this group has demonstrated differences in the sensitivity of a series of human embryo fibroblast cell lines of common genetic origin, to the chemotherapeutic antimetabolites methotrexate (MTX) and *N*-phosphonacetyl-L-aspartate (PALA). This sensitivity could be greatly decreased by the activity of the salvage pathways for purine and pyrimidine biosynthesis (Kinsella and Haran, 1991; Pickard and Kinsella, 1995). A similar pattern of resistance was observed with 5-fluorouracil (5-FU), with the KMS normal cell line being more sensitive than either the KMST or KN-NM cell lines. However, the increased resistance of the KMST and KN-NM cell lines to 5-FU was shown to be unaltered in the presence of the nucleoside transport inhibitor dipyridamole (Pickard and Kinsella, 1995) and thus was independent of salvage pathway involvement.

Progression towards the tumour phenotype results from the interaction of dominantly acting oncogenes and tumour-suppressor genes. It is becoming increasingly clear that the cytotoxic action of many anti-cancer drugs involves processes downstream of the interaction between a particular drug and its target. It has been postulated that the outcome of drug therapy is determined by the response of a particular cell according to its phenotype, rather than by the nature of the primary drug–target interaction alone (Dive and Hickman, 1991).

Alterations in p53 are the most common genetic aberrations found so far in human malignancies (Hollstein *et al.*, 1991; Levine *et al.*, 1991). P53 protein levels are not normally detectable by immunohistochemical techniques, owing to the relatively short half-life of the p53 protein in the cell nucleus (Finlay *et al.*, 1988). It is only when the p53 protein becomes stabilised or overexpressed that the protein becomes detectable immunohistochemically. Stabilised p53 is now thought to reflect a change in the environment of the cell which affects, either directly or indirectly, the p53 protein and indicates an inactivated or impaired function in that particular cell. The tumour-suppressor gene p53 protein product is believed to serve as a critical regulator of a $G_1$ cell cycle checkpoint and as an inducer of apoptosis following exposure of cells to DNA-damaging agents (Katsan *et al.*, 1991; Fritsche *et al.*, 1993; Nelson and Kastan, 1994). DNA damaging agents, including those used in cancer chemotherapy, have been reported to induce nuclear accumulation of wild-type p53 in fibroblasts of both mouse and human origin (Lowe and Ruley 1993; Fritsche *et al.*, 1993), causing $G_1$ arrest, allowing repair processes to operate or, in severe cases of DNA damage, initiation of apoptosis. It has been suggested that DNA strand breaks are the necessary trigger for the p53-dependent DNA damage response pathway (Nelson and Kastan, 1994). Human haematopoietic cells with mutant p53 function do not exhibit $G_1$ arrest after DNA damage and progress through the cell cycle (Kastan *et al.*, 1991; Kuerbitz *et al.*, 1992). Cells with mutant p53 function will continue to proliferate regardless of the insult and will not undergo apoptosis unless mitotic failure occurs as a result of excessive DNA damage (Lane, 1992). Recently immature mouse thymocytes lacking p53 have been shown not to undergo apoptosis when exposed to ionising radiation and demonstrate increased resistance to these lethal effects (Lowe *et al.*, 1993*a*). Studies in certain human tumour cells lacking p53 have also demonstrated increased resistance to ionising radiation as a result of the loss of $G_1$ checkpoint control (McIlwrath *et al.*, 1994; Lee and Bernstein, 1993).

It has been postulated that it is the ability or failure of a cancer cell to undergo apoptosis which is an important determinant of the therapeutic response of that cell to insult (Dive and Hickman, 1991; Lowe *et al.*, 1993*b*). Chemotherapeutic agents including 5-FU along with irradiation and certain hormonal therapies are known to induce apoptosis or programmed cell death in certain cell types (Dive and Hickman, 1991; Lowe *et al.*, 1993*b*). Apoptosis is a genetically controlled cell deletion process that regulates organ development

Correspondence: AR Kinsella
Received 18 April 1995; revised 6 July 1995; accepted 2 August 1995

and tissue maintenance in rapidly proliferating tissues (Raff, 1992) and protects against cancer (Williams, 1991). Genes known to participate in the regulation of apoptosis are E1A (Rao *et al.*, 1992; White *et al.*, 1992; Lowe and Ruley 1993), *myc* (Evan *et al.*, 1992), *bcl*-2 (Hockenberry *et al.*, 1990; Chiou *et al.*, 1994), p53 (Lowe *et al.*, 1993*a*; Clarke *et al.*, 1993) and possibly *ras*.

In the present study we have investigated the difference in resistance observed between the cell lines to 5-FU and tried to establish the basis of this difference. Whether the differences are a result of differing p53 functionality causing differences in apoptosis levels or cell cycle arrest will be discussed along with its implications for malignancy.

### Materials and methods

### *Cell lines*

The cell lines used were the normal human embryo fibroblast cell line KMS-6, its cobalt-60-irradiated immortalised derivative KMST (Namba *et al.*, 1988) and the tumorigenic cell line KN-NM established from the KMST cell line by transfection with an activated N-*ras* oncogene cDNA (Kinsella *et al.*, 1990). This series of cell lines was chosen as a model for the progression of a normal cell towards tumorigenicity as is observed *in vivo*. All cell lines were routinely maintained in monolayer culture in Dulbecco's modified Eagle medium (Life Technologies, Paisley, UK) supplemented with 10% fetal bovine serum (ICN Biomedicals, Irvine, UK) and 200 mM glutamine at 37°C in a humid atmosphere of 5% carbon dioxide/95% air. Cells were routinely plated at a density of $5 \times 10^5$ per 75 $cm^2$ tissue culture flask in 10 ml of medium and passaged approximately every 5 days by trypsinisation.

### *Clonogenic cell survival assays*

To assay for drug resistance (sensitivity) 500 test cells were plated onto a feeder layer of $5 \times 10^4$ gamma-irradiated (5000 rad) EJ human bladder carcinoma cells in a 10 cm tissue culture plate (5 plates per point) and allowed to attach for 4 h. These cell densities were established from preliminary experiments and reflect plating efficiencies over the range of 12.3% (KMS) to 26% (KMST and KN-NM) in control plates. After 4 h the medium was removed and replaced with medium containing 5-FU at the appropriate concentration over the range $1 \times 10^{-7}$ to $1 \times 10^{-4}$ M. The medium was replaced with drug-free medium after weeks 1 and 2. The plates were incubated for a total of 3 weeks to permit colony formation. After 3 weeks the medium was removed and any colonies present were fixed in ice-cold methanol for 15 min and stained with 10% Giemsa stain. Colonies were counted and the colonies on the treated plates were expressed as a percentage of the colonies on control plates to which no drug had been added. These assays were not designed to try and recreate conditions experienced *in vivo* in the clinic. The exposure of the cells to drug for 1 week was longer than the exposure experienced by a cell *in vivo* and the doses used were at times superphysiological. With these assays an attempt was made to understand the basic mechanisms occurring in the cell which may be causing the difference in resistance observed. This particular assay was carried out on three separate occasions.

### *Immunohistochemical analysis for P53 protein*

Asynchronous log phase cultures of the KMS, KMST and KN-NM cell lines were harvested and $1 \times 10^7$ cells were resuspended in 10% formalin solution for 4 h. The cells were then pelleted and embedded in agar before being placed in a tissue cassette for tissue processing. The pellets were processed overnight before being paraffin embedded. Sections (4 μm) were then cut on a microtome and mounted on poly-L-lysine-coated microscope slides. The sections were then dewaxed in xylene, dehydrated in alcohol and microwaved for 15 min in citrate buffer pH 6.0 to aid antigen retrieval. Any endogenous peroxidase activity was blocked by immersing the slides in 3% hydrogen peroxide solution for 30 min. The Ab6 (Oncogene Science) mouse DO1 clone (Vojtesek *et al.*, 1992) anti-p53 antibody diluted to 2 $\mu g\ ml^{-1}$ in Tris-buffered saline (TBS) was then applied to each section and incubated at room temperature for 1 h. With $2 \times 5$ min washes in TBS between each step the following reagents were applied to the sections for the indicated lengths of time: goat anti-mouse biotinylated secondary antibody, 30 min (Vectastain); ABC avidin–biotin complex, 30 min (Vectastain); DAB solution, 10 min; Meyer's haemotoxylin solution, 30 s. Slides were mounted with DPX and viewed under a light microscope. Brown nuclear coloration indicated elevated levels of p53 protein.

### *Assessment of cell growth and apoptosis*

Test cells were plated at a density of $5 \times 10^5$ per 75 cm tissue culture flask. Two flasks were seeded per drug concentration. The medium was removed 24 h later and replaced with the corresponding drug-containing medium at the relevant drug concentrations. Every 24 h thereafter, the medium was removed and spun at 1500 r.p.m. for 5 min to pellet any detached cells before being returned to its respective flask for further incubation. The 'detached cell pellet' was resuspended in 20 μl of medium. A 4 μl sample of this suspension was taken and added to 4 μl of trypan blue and placed on a haemocytometer counting chamber to obtain a count of the number of detached cells. Another 4 μl sample was added to 4 μl of acridine orange on a microscope slide and viewed under a fluorescent microscope at an exciting wavelength of 490 nM (Evans and Dive, 1993). Five horizontal fields were viewed and the number of cells identified as apoptotic, viable and 'ghost' were counted. The number of apoptotic cells was expressed as a percentage of all the detached cells and this figure was used to adjust the haemocytometer cell count to give a final estimation of the total number of apoptotic cells in that sample. The ghost cell count was ignored in the final calculation because, although these cells had most likely been through the process of apoptosis, they were not at the time of measurement and hence not a true reflection of the level of apoptosis at that time.

Twenty flasks per cell line were run in parallel in order to determine the growth curve for the same cell population over the measured period and to enable the number of apoptotic cells at each dose to be expressed as a percentage of the total cell population. These flasks were incubated in identical conditions and trypsinised for counting every 48 h from time 0 to day 8 of the experiment. All experiments were carried out on at least three separate occasions and the results presented are of data from one representative experiment.

### *Measurement of cell cycle distribution*

The cell cycle distributions of the trypsinised monolayers (but not detached cells) were measured on days 3 and 6 of the experiment. Cell samples for flow cytometry were fixed in 1% paraformaldehyde and 1% Triton X-100 for 10 min before being washed and resuspended in phosphate-buffered saline (PBS). Propidium iodide (PI) (20 μl of a 2.5 $mg\ ml^{-1}$ solution in PBS) and RNAse (10 μl of a 10 $mg\ ml^{-1}$ solution) were added to $5 \times 10^5$ cells 500 $\mu l^{-1}$ PBS for 30 min at 37°C. The position of cells in the cell cycle can be estimated as a function of their DNA content. Propidium iodide was used to stain the DNA of 20 000 cells and the red fluorescence produced on excitation with an argon ion laser was analysed using a Coulter Epics XL flow cytometer. Cytograms of DNA peak *vs* the area of DNA signal were produced for each cell sample. The single cell population was enclosed in a gate and from these data DNA histograms were generated. Cells in $G_1$, as well as $G_2 + M$ cells, have DNA contents, equivalent to DNA ploidy indices 1.0 and 2.0 respectively. The DNA content doubles during S-phase of the cell cycle and therefore an estimation of the number of cells between the $G_1$ and $G_2/M$ peaks gives an indication of the number of cells passing through the S-phase. Regions were assigned to each histogram which were thought to best represent the $G_1$, S- and $G_2/M$ phases for each cell line. From these regions the percentage of cells in each phase of the cell cycle was calculated. The position of the cytogram gates and the histogram regions were set on the control sample for each cell line

**Figure 1** Immunohistochemical localisation of p53 protein in the series of human embryo fibroblast cell lines. Cell pellets were fixed in formalin, embedded in paraffin and cut into 4 μM sections. Sections were stained for p53 protein with the Ab6 DO1 antibody (Oncogene Science) and detected using a biotinylated secondary antibody and avidin–biotin immunoperoxidase detection system as recommended by the manufacturer (Vectastain). Negative control sections were prepared by omitting the p53 antibody to assess background peroxidase staining. (**a**) KMS normal fibroblast cell line with antibody. (**b**) KMS without antibody. (**c**) KMST immortalised fibroblast cell line with antibody. (**d**) KMST without antibody. (**e**) KN-NM tumorigenic cell line with antibody. (**f**) KN-NM without antibody.

and were not altered for any of the subsequent drug-treated cell samples. This experiment was carried out on at least three separate occasions and at various other time points after drug addition. The data presented are from one representative experiment.

### Results

### *P53 status of the three cell lines*

The ABC peroxidase staining method was used to stain any p53 protein present in the cell nucleus (Figure 1). Brown coloration in the nucleus indicated the presence of p53 protein. Overexpression or stabilisation of the p53 protein results in levels that are detectable with immunohistochemistry. (Finlay *et al.*, 1988; Gannon *et al.*, 1990; Iggo *et al.*, 1990). Apart from slight background staining, the KMS cell line showed no signs of p53 protein overexpression, suggesting that this cell line possessed wild-type p53 function (Figure 1a and b). This was in contrast to both the KMST and KN-NM cell lines which showed definite brown nuclear staining, indicating p53 overexpression/stabilisation and possible disruption of p53 wild-type function (Figure 1c–f).

### *Effect of different 5-FU drug concentration on cell survival*

For all three cell lines the colonies that survived the increasing concentrations of 5-FU were scored after 3 weeks of incubation, expressed as a percentage of the number of colonies on control drug-free plates and plotted against increasing drug concentration. It can be observed that the normal KMS cell line is more sensitive to 5-FU than the KMST or KN-NM cell line (for representative data from one experiment see Figure 2). No difference in resistance was apparent between the KMST and the KN-NM cell lines.

### *Growth of cell lines in the presence of increasing concentrations of 5-FU*

The differences in resistance observed in the colony-forming assay were supported by the growth curves obtained for each of the cell lines at the three different drug concentrations (for representative data from one experiment see Figure 3a, b and c). The normal KMS cell line in control drug-free medium proliferated in a normal fashion with a doubling time of around 48 h and reached confluence by days 7–8 with a saturation cell density of $5.5 \times 10^6$ cells. At drug concentrations of $1 \times 10^{-5}$ M and $1 \times 10^{-4}$ M 5-FU no proliferation or cell detachment was apparent and the cell number stayed at a constant level until day 8 at approximately $5 \times 10^5$ cells. At a drug concentration of $1 \times 10^{-3}$ M 5-FU the cell number stayed constant until approximately day 4 and then began to decrease, reaching a negligible cell level by day 8. The immortalised KMST cell line reached a saturation cell density of $7.4 \times 10^6$ cells by day 8. Unlike KMS, growth of the KMST cell line was apparent at a drug concentration of $1 \times 10^{-5}$ M 5-FU. As with KMS, the KMST population appeared to remain static at a drug concentration of $1 \times 10^{-4}$ M. At a drug concentration of $1 \times 10^{-3}$ M the population number decreased after day 4 to reach a negligible level by day 8. The tumorigenic, KN-NM, N-*ras*-transformed cell line showed the highest level of proliferation of all three cell lines, with a doubling time of approximately 36 h. However, no difference in resistance was apparent when compared with the KMST cell line (Figure 2). The growth rate of the KN-NM cell line was rapid, reaching a saturation cell density of approximately $1.2 \times 10^7$ cells by day 6. Increased growth, in terms of increased cell numbers, was apparent when com-

**Figure 2** Intrinsic 5-FU sensitivities of the three different cell lines in DMEM supplemented with 10% fetal calf serum. ●, KMS normal fibroblasts; ◆, KMST immortalised fibroblasts; ▲, KN-NM tumorigenic cells. These data are taken from one representative experiment. Each point represents the mean of five plates.

**Figure 3** Growth curves for the three cell lines in the presence of differing concentrations of 5-FU. (**a**) KMS normal fibroblasts. (**b**) KMST immortalised fibroblasts. (**c**) KN-NM tumorigenic fibroblasts. □, Control; ○, $1 \times 10^{-5}$ M; ▲, $1 \times 10^{-4}$ M; △, $1 \times 10^{-3}$ M, 5-FU. Results shown are of one representative repeat experiment out of three.

pared with the KMST cell line at a drug concentration of 1 × $10^{-5}$ M 5-FU. At 1 × $10^{-4}$ M 5-FU, as with the KMS and KMST cell lines, there was no increase in population size and a concentration of 1 × $10^{-3}$ M resulted in a decrease in population size after day 4 to negligible cell levels by day 8.

These data clearly demonstrate the differences in resistance of the three cell lines to 5-FU administration. The major difference can be observed (Figure 3) at a dose of 1 × $10^{-5}$ M 5-FU with no growth of the normal KMS cell line in comparison with growth of the immortalised KMST and the tumorigenic KN-NM cell lines. Repeated experiments consistently demonstrated this pattern.

### *Levels of apoptosis in the three cell lines*

Following initial experiments to establish the levels and rates of cell death, apoptosis was measured every 24 h for all three cell lines for a period of 8 days following drug addition. The drug was not removed from the medium at any time during this period. The control untreated cell populations showed levels of apoptosis ranging from negligible at <0.15% of the total cell population in the KMS cell line to higher levels of 1.5 and 2.5% for KMST and KN-NM cell lines respectively (Figure 4). High levels of apoptosis were observed in all three cell lines 6 days after continuous exposure to the highest drug concentrations of 1 × $10^{-3}$ M 5-FU, at levels of 52% of the total cell population in KMS, 51% in KMST and 42% in KN-NM (data from one representative experiment). However, a difference in the level of apoptosis was observed between the KMS normal cell line and the KMST and KN-NM cell lines in response to lower concentrations of 5-FU. At drug concentrations of 1 × $10^{-5}$ and 1 × $10^{-4}$ M, the KMS cell line showed negligible levels of apoptosis (Figure 4). These levels were in contrast to those seen in the KMST and KN-NM cell lines, at the same drug concentrations, which both demonstrated increasing levels of apoptosis through the 8 day period. The level of apoptosis appeared to increase in a dose-dependent manner with both the KMST and KN-NM cell lines reaching levels of 8.2% and 17.8% respectively, by day 8 at a concentration of 1 × $10^{-4}$ M (Figure 4).

**Figure 4** Levels of apoptosis expressed as a percentage of the total cell population. (**a**) KMS normal fibroblasts. (**b**) KMST immortalised fibroblasts. (**c**) KN-NM tumorigenic fibroblasts. □, Control; ○, 1 × $10^{-5}$ M; ▲, 1 × $10^{-4}$ M, 5-FU. The levels of apoptosis at a concentration of 1 × $10^{-3}$ M 5-FU have been omitted from the graph as this dose appeared to be universally toxic and induced high levels of apoptosis in all the cell lines. Results shown are of one representative repeat experiment out of three.

### *Effect on cell cycle*

The DNA content of 20 000 cells was measured. The cytograms and histograms for the three cell lines representing the state of their cell cycles 3 days after continuous exposure to various concentrations of 5-FU are shown in Figure 5. The percentage of cells in each phase of the cell cycle are contained in Table I. A time point of 3 days was established from preliminary experiments and was chosen because of the relatively slow doubling time of the cell lines and to allow for the delay in action required by 5-FU. With increasing 5-FU concentrations up to a level of 1 × $10^{-4}$ M, the KMS cell line maintained a definite $G_1/G_0$ arrest, displaying only a slight fall in percentage from 81.7% (control) to 76.8%. The percentage of cells in $G_2/M$ phase rose in a dose-dependent manner reaching a level of 34.8% at 1 × $10^{-3}$ M, while the number of cells in S-phase decreased from 7.1% at control level to just 2.4% at a 5-FU concentration of 1 × $10^{-3}$ M. This was in contrast to the situation with the KMST and KN-NM cell lines, which both demonstrated large increases in the number of cells in S-phase with increasing 5-FU concentration while the number of cells in $G_1/G_0$ and $G_2/M$ decreased. The percentage of cells in S-phase rose from a control level of 13.8 and 7.2 to 37.3 and 29.8 at a drug concentration of 1 × $10^{-3}$ M, for the KMST and KN-NM cell lines respectively.

### Discussion

Previous studies from this group have shown that differences in the resistance of a series of human embryo fibroblasts to MTX and PALA can be greatly exaggerated by the involvment of the salvage pathways for purine and pyrimidine biosynthesis (Kinsella and Haran, 1991; Pickard and Kinsella, 1995). A similar pattern of increasing resistance paralleling increasing tumorigenicity was observed for 5-FU, but as stated previously, was independent of salvage pathway involvement. No change in the resistance to 5-FU was observed in any of the three cell lines in the presence of the dipyridamole at a concentration of 5 μM. Dipyridamole has previously been shown to be an effective inhibitor of nucleoside transport (Berlin and Oliver, 1972; Paterson *et al.*, 1980; Plagemann and Wolhueter, 1980; Young and Jarvis, 1983; Zhen *et al.*, 1983). A significant increase in the sensitivity of the KMST and KN-NM cell lines was observed when MTX and PALA were administered in the presence of dipyridamole (Pickard and Kinsella, 1995).

Immunohistochemical analysis revealed differences in the levels of p53 protein between the three cell lines. These data suggested that wild type-p53 function may have been disrupted in the immortalised KMST and the tumorigenic KN-NM cell lines (Figure 1). Increased p53 protein stability in a

**Figure 5** Cell cycle changes in the three cell lines after 3 days of exposure to increasing concentrations of 5-FU. The $G_1/G_0$-,S- and $G_2/M$- phases of the cell cycle are defined on the histograms and can be observed on the cytograms (inset) as $G_1/G_0$ (lower aggregation), $G_2/M$ (upper aggregation) and S as the area in between. The percentage of cells in each phase are presented in Table I

Table I Cell cycle data for the 3 cell lines after 72 h of continuous exposure to the various concentrations of 5-FU

| Cell line | 5-FU conc. (M) | Cell cycle phase % Go/G1 | %S | %G2 + M |
|---|---|---|---|---|
| KMS (Normal) | Control | 81.7 | 7.1 | 13.1 |
| | $1 \times 10^{-5}$ | 83.5 | 1.4 | 14.5 |
| | $1 \times 10^{-4}$ | 76.8 | 1.8 | 20.5 |
| | $1 \times 10^{-3}$ | 62.7 | 2.4 | 34.8 |
| KMST | Control | 60.5 | 13.8 | 25.3 |
| (Immortal) | $1 \times 10^{-5}$ | 56.6 | 12.7 | 29.9 |
| | $1 \times 10^{-4}$ | 38.4 | 40.7 | 20.0 |
| | $1 \times 10^{-3}$ | 44.4 | 37.3 | 22.5 |
| KN-NM | Control | 65.9 | 7.2 | 26.4 |
| (Tumorigenic) | $1 \times 10^{-5}$ | 67.0 | 10.7 | 23.2 |
| | $1 \times 10^{-4}$ | 56.0 | 29.4 | 14.0 |
| | $1 \times 10^{-3}$ | 53.8 | 29.8 | 19.6 |

cell nucleus was thought, until recently, to correlate directly with mutation of the p53 gene (Gannon *et al.*, 1990; Iggo *et al.*, 1990; Baas *et al.*, 1994). This association has recently been called into question following the publication of a number of studies suggesting false-positive and negative results (Borreson *et al.*, 1991; Cripps *et al.*, 1994). It has now been reported that, as well as mutation, other factors can stabilise p53 protein and render it non-functional (Vogelstein and Kinzler, 1992). Interactions with viral proteins, such as the large T antigen of SV40 (Lane and Benchimol, 1990; Levine *et al.*, 1991), or interactions with cellular proteins such as the *mdm*-2 gene product (Wu *et al.*, 1993) have been shown to stabilise p53 protein. These observations have now led to the theory that it is the whole cellular environment that determines p53 stability and therefore function (Hall and Lane, 1994). These data suggest that stabilised, immunohistochemically detectable p53 protein, whether created as a result of mutation or by some other protein interaction, may have an inactivated or impaired function in the cell. The p53 tumour-suppressor gene product is now thought to play an important role as a $G_1$ to S checkpoint control in the cell cycle and as a controller of cells entering apoptosis, following DNA damage (Kastan *et al.*, 1991; Kuerbitz *et al.*, 1992). DNA strand breaks are thought to be an important factor in initiating this DNA damage response pathway (Nelson and Kastan, 1994). It has been postulated that the outcome of drug therapy will be determined by the response of the cell, according to its phenotype, rather than by the nature of the primary drug target interaction alone (Dive and Hickman, 1991). The response of the cell can manifest itself in several ways including apoptosis (Clarke *et al.*, 1993; Lowe *et al.*, 1993*b*, *c*), cell cycle arrest (McIlwrath *et al.*, 1994; Nelson and Kastan, 1994) or drug-induced increases in metastatic potential (McMillan and Hart, 1987).

This, together with the evidence for differences in p53 functionality between the cell lines, lead us to postulate that the differences in cell line sensitivity to 5-FU (Figure 2) may be the result of differing cellular responses to drug-induced damage. Contrary to expectation, measurement of apoptosis in the three cell lines showed the normal KMS cell line, with wild-type p53 protein levels, to apoptose at a lower level than the more resistant KMST and KN-NM cell lines. From these data it became clear that the differences in the levels of apoptosis between the cell lines could not explain the differences in resistance observed.

Detailed cell cycle analysis showed that the KMS cell line ceased to proliferate in response to increasing levels of 5-FU and appeared to be in some type of growth arrest. This was in contrast to both the KMST and KN-NM cell lines, which appeared to continue proliferating regardless of the insult. Representative cytograms and DNA histograms obtained for the three cell lines after 3 days' exposure to increasing concentrations of 5-FU can be observed in Figure 5. This time point was independent of any influence of growth factor depletion or contact inhibition on the cell proliferation rate, as can be observed from the growth curves (Figure 3). All three cell lines showed classical cell cycle patterns in control drug-free medium, with a $G_1$ and $G_2$ peak divided by cells in S-phase. The KMS normal cell line showed a decrease in the percentage of cells in S-phase with increasing 5-FU concentration, with a $G_1$ arrest predominating at low drug concentrations and a $G_2$/M arrest at higher drug concentrations. This type of response to presumed drug-induced DNA damage is the classical pattern for wild-type p53 function as first described by Kastan *et al.* (1991). Cells that are already in S-phase at the time of DNA damage continue to progress through to $G_2$/M, whereas cells in $G_1$ do not continue to enter S-phase. After 1 week of exposure the drug was removed from the cells. The KMS cells exposed to the two highest doses of 5-FU showed no signs of further proliferation and were confirmed to be in permanent growth arrest. One may predict that the amount of damage in these cells was at such a high level that the cells were held in a permanent state of growth arrest. Leonardo *et al.* (1994) have recently reported a similar permanent growth arrest response in human fibroblasts following ionising radiation. Thus, in addition to apoptosis and transient cell cycle arrest, permanent growth arrest may be a third type of cellular damage response. The immunohistochemical and cell cycle data are consistent with the KMS cell line having wild-type p53 function. This is in contrast to the situation seen with both the KMST and KN-NM cell lines. These cell lines demonstrated a dramatic increase in the percentage of cells in S-phase with increasing concentrations of 5-FU. The number of cells in both $G_1$ and $G_2$/M decreased with increasing 5-FU concentrations. These data, along with the KMST and KN-NM immunohistochemical data, therefore suggest that wild-type p53 function may have been lost from both these cell lines and that, especially at high doses of 5-FU, the cell cycle is well out of control.

The effect of the activated N-*ras* oncogene on the growth rate of the KN-NM cell line was easily recognisable (Figure 3). Growth was much more rapid and the cell number reached a much higher level in 8 days than the KMST cell line. Although proliferation was increased and colony formation was more substantial in the tumorigenic KN-NM cell line this appeared to have no effect on the overall resistance of the cell population to 5-FU (Figure 2).

In conclusion, these data have demonstrated an association between cell cycle changes and sensitivity to 5-FU. Normal cells appear to modulate this response by a permanent $G_1$ arrest, with significant apoptosis only occurring at the highest doses of 5-FU. Contrary to expectation, the cells tending towards tumorigenicity underwent higher levels of apoptosis than the normal cells. This phenomenon may have been related to increasing levels of 5-FU and presumably DNA damage in cells lacking cell cycle control, resulting in spontaneous apoptosis as a result of a failure in mitosis. In this series of experiments, p53 was not artificially inserted into the cell lines, however both indirect immunohistochemical and direct cell cycle evidence suggests that wild-type p53 function has been lost by the cells tending towards tumorigenicity. This model is thought to be a good reflection of the situation actually occurring *in vivo*, as a cell progresses from its normal phenotype to one of a tumorigenic cell. These data demonstrate the effect of loss of cell cycle control, whether p53 dependent or not, in regulating the sensitivity of human cells to DNA-damaging agents. If cell cycle control is missing, as in the progression towards malignancy, then increasingly resistant phenotypes appear to result. Clearly the next experiments need to be conducted with the use of cells of known p53 status, employing the use of wild-type and mutant p53-containing vectors.

### Abbreviations
MTX, methotrexate; 5-FU, 5-fluorouracil; PALA, *N*-phosphonacetyl-L-aspartate

### Acknowledgements
The authors would like to thank Mr J Culkin and Mr R Brew, Department of Immunology, Liverpool University and Mrs Bindy Heer, Department of Physiological Sciences, Manchester University for all their valuable help with the flow cytometry analysis. This work was funded by an NWCRF grant to Anne R Kinsella.

### References

BAAS IO, MULDER JWR, OFFERHAUS JA, VOGELSTEIN B AND HAMILTON SR. (1994). An evaluation of six antibodies for immunohistochemistry of mutant p53 gene product in archival colorectal neoplasms. *J. Pathol.*, **172,** 13–18.

BERLIN RD AND OLIVER JM. (1972). Membrane transport of purine and pyrimidine bases and nucleosides in animal cells. *Int. Rev. Cytol.*, **42,** 287–336.

BORRESON AL, HOVIG E AND SMITH-SORENSEN B. (1991). Constant denaturant gel electrophoresis as a rapid screening technique for p53 mutations. *Proc. Natl Acad. Sci. USA*, **88,** 8405–8409.

CHIOU SK, RAO L AND WHITE E. (1994). Bcl-2 blocks p53-dependent apoptosis. *Mol. Cell. Biol.*, **14,** 2556–2563.

CLARKE AR, PURDIE CA, HARRISON DJ, MORRIS RG, BIRD CC, HOOPER ML AND WYLLIE AH. (1993). Thymocyte apoptosis induced by p53-dependent and independent pathways. *Nature*, **362,** 849–852.

CRIPPS KJ, PURDIE CA, CARDER PJ, WHITE S, KOMINE K, BIRD CC AND WYLLIE AH. (1994). A study of stabilisation of p53 protein versus point mutation in colorectal carcinoma. *Oncogene*, **9,** 2739–2743.

DIVE C AND HICKMAN JA. (1991). Drug–target interactions: only the first step in the commitment to a programmed cell death? *Br. J. Cancer*, **64,** 192–196.

EVAN GI, WYLLIE AH, GILBERT CS, LITTLEWOOD TD, LAND H, BROOKS M, WATERS CM, PENN LZ AND HANCOCK DC. (1992). Induction of apoptosis in fibroblasts by c-myc protein. *Cell*, **69,** 119–128.

EVANS DL AND DIVE C. (1993). Effects of cisplatin on the induction of apoptosis in proliferating hepatoma cells and nonproliferating immature thymocytes. *Cancer Res.*, **53,** 2133–2139.

FINLAY CA, HINDS PW, TAN T-H, ELIYAHU D, OREN M AND LEVINE AJ. (1988). Activating mutations for transformation by p53 produce a gene product that forms an hsp 70–p53 complex with an altered half life. *Mol. Cell. Biol.*, **8,** 531–539.

FOX M, BOYLE JM AND KINSELLA AR. (1991). Nucleoside salvage and resistance to antimetabolite anticancer agents. *Br. J. Cancer*, **64,** 428–436.

FRITSCHE M, HAESSLER C AND BRANDNER G. (1993). Induction of nuclear accumulation of the tumour suppressor protein p53 by DNA-damaging agents. *Oncogene*, **8,** 307–318.

GANNON JV, GREAVES R, IGGO R AND LANE DP. (1990). Activating mutations in p53 produce a common conformational effect. A monoclonal antibody specific for the mutant form. *EMBO J.*, **9,** 1595–1602.

HALL PA AND LANE DP. (1994). P53 in tumour pathology: Can we trust immunohistochemistry? – Revisited. *J. Pathol*, **172,** 1–4.

HOCKENBERRY D, NUNEZ G, MILLIMAN C, SCHREIBER RD AND KORSMEYER S. (1990). Bcl-2 is an inner mitochondrial membrane protein that blocks programmed cell death. *Nature*, **348,** 334–336.

HOLLSTEIN M, SIDRANSKY D, VOGELSTEIN B AND HARRIS CC. (1991). P53 mutations in human cancers. *Science*, **253,** 49–52.

IGGO R, GATTER K, BARTEK J, LANE DP AND HARRIS A. (1990). Increased expression of mutant forms of p53 oncogene in primary lung cancer. *Lancet*, **335,** 675–679.

KASTAN MB, ONYEKWERE O, SIDRANSKY D, VOGELSTEIN B AND CRAIG RW. (1991). Participation of p53 in the cellular response to DNA damage. *Cancer Res.*, **51,** 6304–6311.

KINSELLA AR, FISZER-MALISZEWSKA L, MITCHELL ELD, GUO Y, FOX M AND SCOTT D. (1990). Introduction of the activated N-*ras* oncogene into human fibroblasts by retroviral vector induces morphological transformation and tumorigenicity. *Carcinogenesis*, **11,** No. 10, 1803–1809.

KINSELLA AR AND HARAN MS. (1991). Decreasing sensitivity to cytotoxic agents parallels increasing tumorigenicity in human fibroblasts. *Cancer Res.*, **51,** 1855–1859.

KUERBITZ SJ, PLUNKETT BS, WALSH WV AND KASTAN MB. (1992). Wild-type p53 is a cell cycle checkpoint determinant following irradiation. *Proc. Natl Acad. Sci. USA*, **89,** 7491–7495.

LANE DP AND BENCHIMOL S. (1990). P53: Oncogene or antioncogene? *Genes Dev.*, **4,** 1–8.

LANE DP. (1992). p53, guardian of the genome. *Nature*, **358,** 15–16.

LEE JM AND BERNSTEIN A. (1993). P53 mutation increases resistance to ionising radiation. *Proc. Natl Acad. Sci. USA*, **90,** 5742–5746.

LEONARDO AD, LINKE SP, CLARKIN K AND WAHL GM. (1994). DNA damage triggers a prolonged p53-dependent $G_1$ arrest and long-term induction of Cip1 in normal human fibroblasts. *Genes Dev.*, **8,** 2540–2551.

LEVINE AJ, MOMAND J AND FINLAY CA. (1991). The p53 tumour suppressor gene. *Nature*, **351,** 453–456.

LOWE SW AND RULEY EH. (1993). Stabilization of the p53 tumor suppressor is induced by adenovirus 5 E1A and accompanies apoptosis. *Genes Dev.*, **7,** 535–545.

LOWE SW, SCHMITT EM, SMITH SW, OSBORNE BA AND JACKS T. (1993*a*). P53 is required for radiation-induced apoptosis in mouse thymocytes. *Nature*, **362,** 847–849.

LOWE SW, RULEY EH, JACKS T AND HOUSMAN DE. (1993*b*). P53-dependent apoptosis modulates the cytotoxicity of anticancer agents. *Cell*, **74,** 957–967.

MCILWRATH AJ, VASEY PA, ROSS GM AND BROWN R. (1994). Cell cycle arrests and radiosensitivity of human tumour cell lines: Dependence on wild-type p53 for radiosensitivity. *Cancer Res.*, **54,** 3718–3722.

MCMILLAN TJ AND HART IR. (1987). Can cancer chemotherapy enhance malignant behaviour of tumours? *Can Met. Rev.*, **6,** 503–520.

NAMBA M, NISHITANI K, FUKUSHIMA F AND KIMOTO T. (1988). Multi-step carcinogenesis of normal human fibroblasts. *Anticancer Res.*, **8,** 947–958.

NELSON WG AND KASTAN MB. (1994). DNA strand breaks: the DNA template alterations that trigger p53-dependent DNA damage response pathways. *Mol. Cell. Biol.*, **14,** 1815–1823.

PATERSON ARP, LAU EY, DAHLIG E AND CASS CE. (1980). A common basis for inhibition of nucleoside transport by dipyridamole and nitrobenzylthionosine? *Mol. Pharmacol.*, **18,** 40–44.

PICKARD M AND KINSELLA AR. (1995). Reduction in antimetabolite drug resistance by dipyridamole salvage pathway blockade and dependence on cellular response. *Biochem. Pharmacol.* (submitted).

PLAGEMANN PG AND WOHLHUETER RM. (1980). Permeation of nucleosides, nucleic acid bases and nucleotides in animal cells. *Current Topics Membrane Transport*, **14,** 225–230.

RAFF MC. (1992). Social controls on cell survival and cell death. *Nature*, **356,** 397–400.

RAO L, DEBBAS M, SABBATINI P, HOCKENBERRY D, KORSMEYER S AND WHITE E. (1992). The adenovirus E1A proteins induce apoptosis which is inhibited by the E1B 19K and Bcl-2 proteins. *Proc. Natl Acad. Sci. USA*, **89,** 7742–7746.

SCHIMKE RT, ALT FW, KELLEMS RE, KAUFMAN RJ AND BERTINO JR. (1977). Amplification of dihydrofolate reductase genes in methotrexate-resistant cultured mouse cells. *Cold Spring Harbor Symp. Quant. Biol*, **42,** 649.

VOGELSTEIN B AND KINZLER KW. (1992). P53 function and dysfunction. *Cell*, **70,** 523–526.

VOJTESEK B, BARTEK J, MIDGLEY CA AND LANE DP. (1992). An immunochemical analysis of the human nuclear phosphoprotein p53: new monoclonal antibodies and epitope mapping using recombinant p53. *J. Immunol. Methods*, **151,** 237–244.

WAHL GM, PAGGET RA AND STARK GR. (1979). Gene amplification causes over production of the first three enzymes of UMP synthesis in N-(phosphonacetyl)-L-aspartate-resistant hamster cells. *J. Biol. Chem.*, **254,** 8679.

WEBER G. (1983). Biochemical strategy of cancer cells and the design of chemotherapy. G.H.A. Clowes Memorial Lecture. *Cancer Res.*, **43,** 3466.

WHITE E, SABBATINI P, DEBBAS M, WOLD WM, KUSHER DI AND GOODING LR. (1992). The 19-Kilodalton adenovirus E1B transforming protein inhibits programmed cell death and prevents cytolysis by tumour necrosis factor alpha. *Mol. Cell. Biol.*, **12,** 2570–2580.

WILLIAMS GT. (1991). Programmed cell death: Apoptosis and oncogenesis. *Cell.*, **65,** 1097–1098.

WU X, BAYLE H, OLSON D AND LEVINE AJ. (1993). The p53-mdm-2 autoregulatory feedback loop. *Genes Dev.*, **7,** 1126–1132.

YOUNG JD AND JARVIS SM. (1983). Nucleoside transport in animal cells. *Review. Biosci. Res.*, **3,** 309–322.

ZHEN Y-S, LUI MS AND WEBER G. (1983). Effects of acivicin and dipyrimadole on hepatoma 3924A cells. *Cancer Res.*, **43,** 1616–1619.


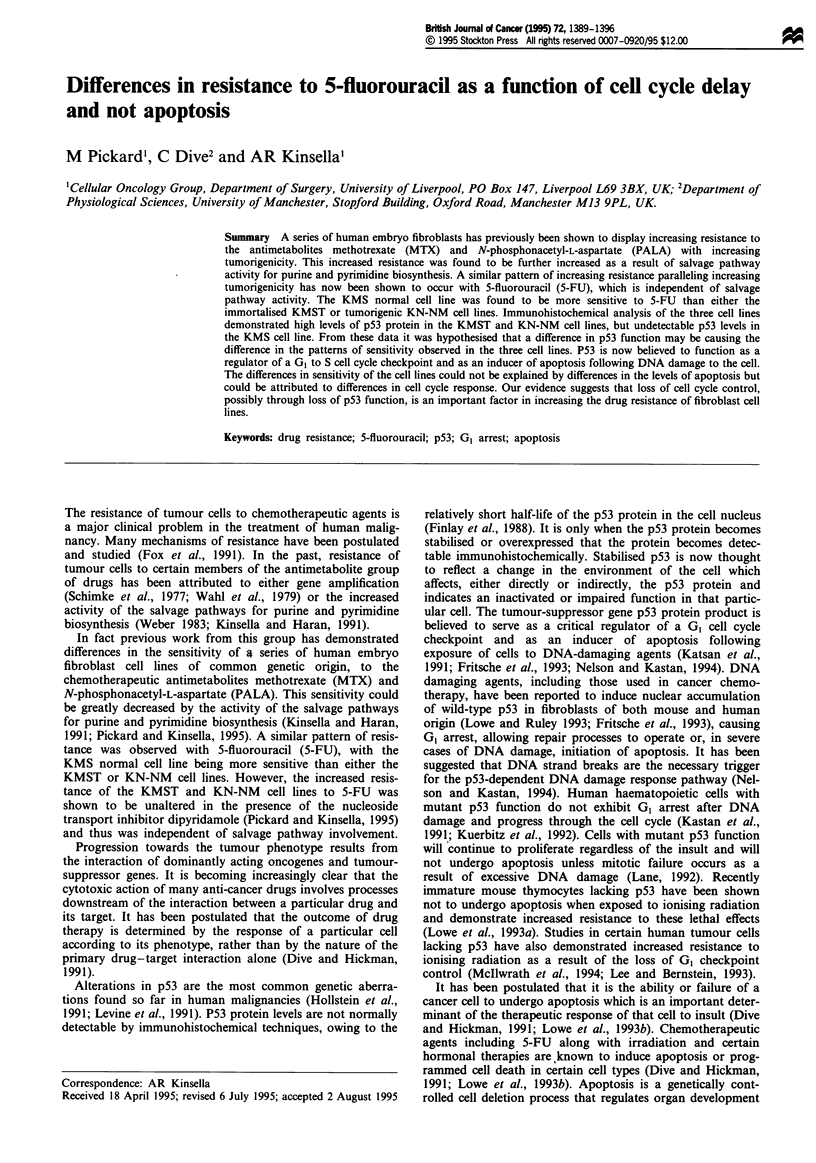

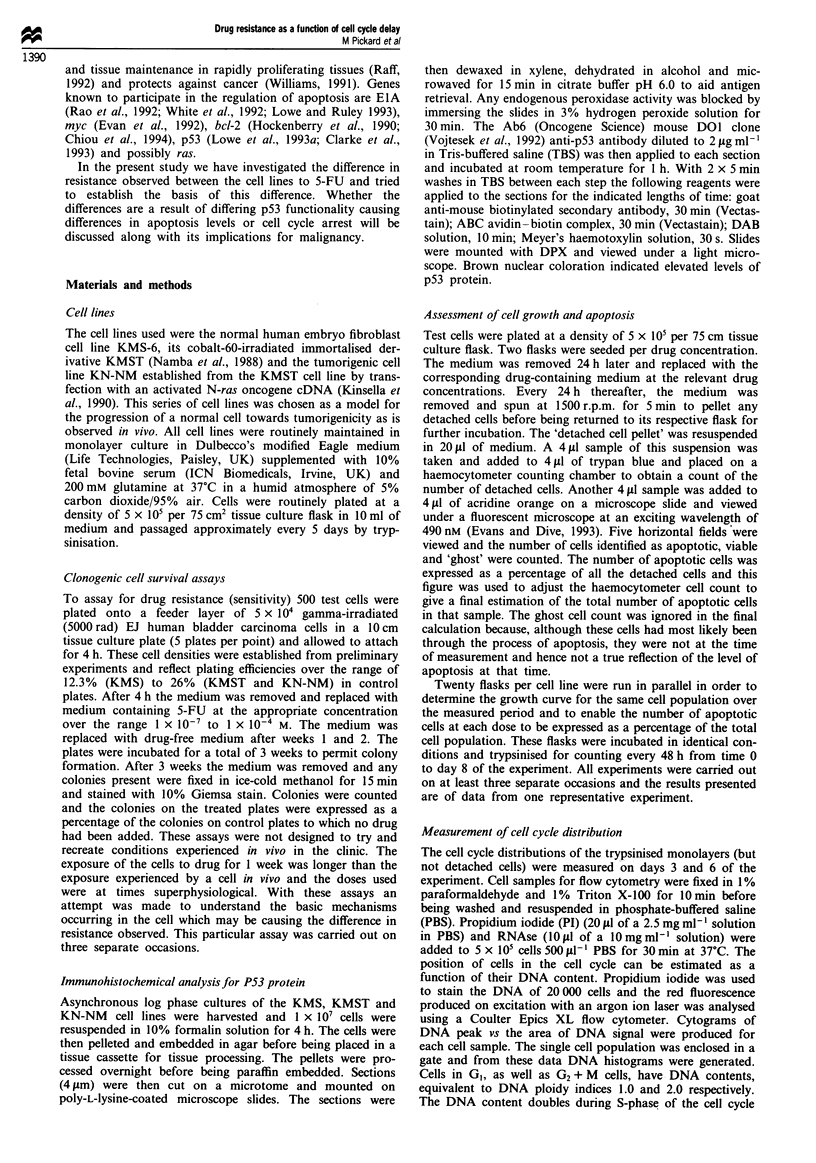

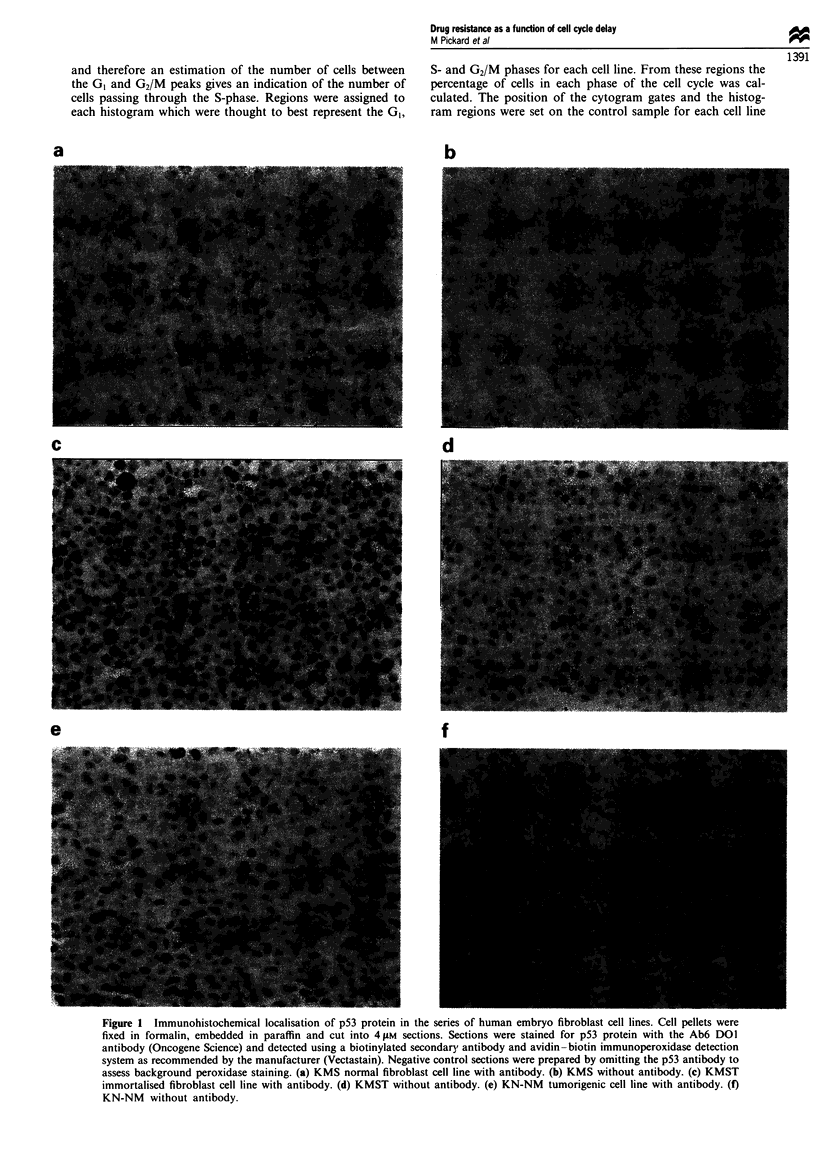

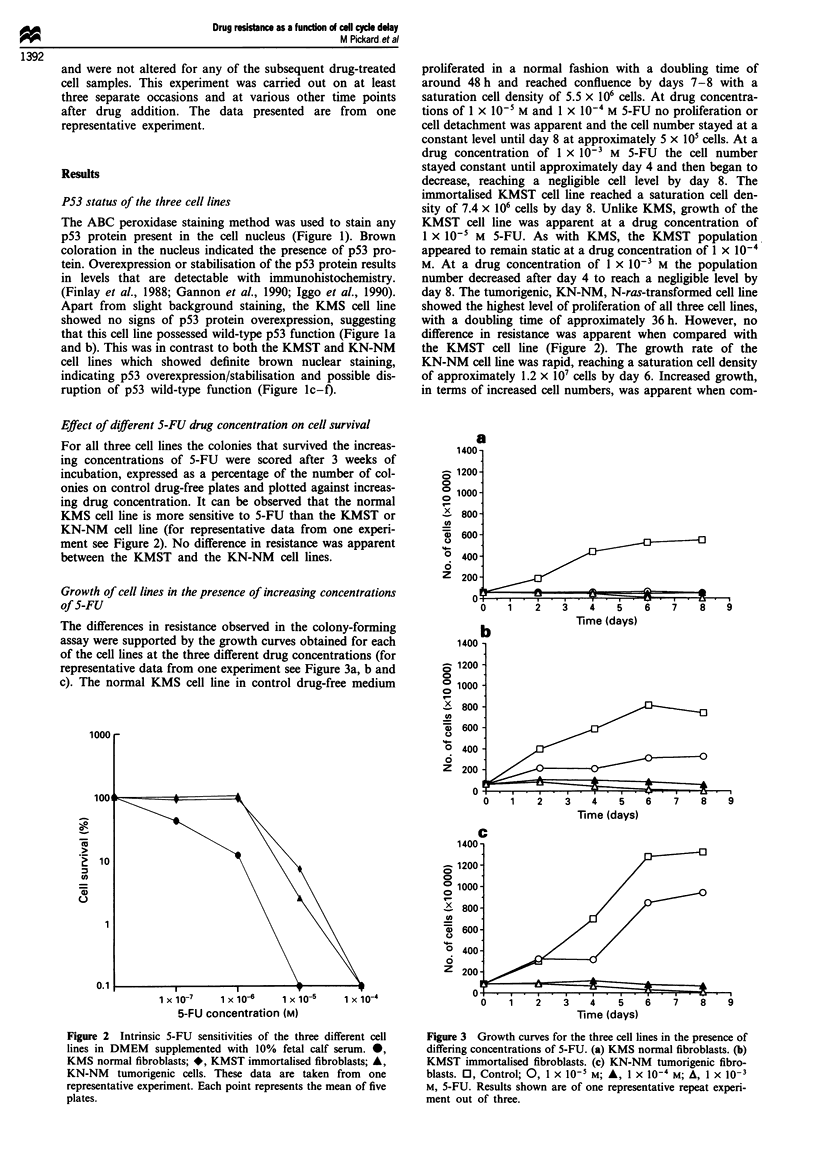

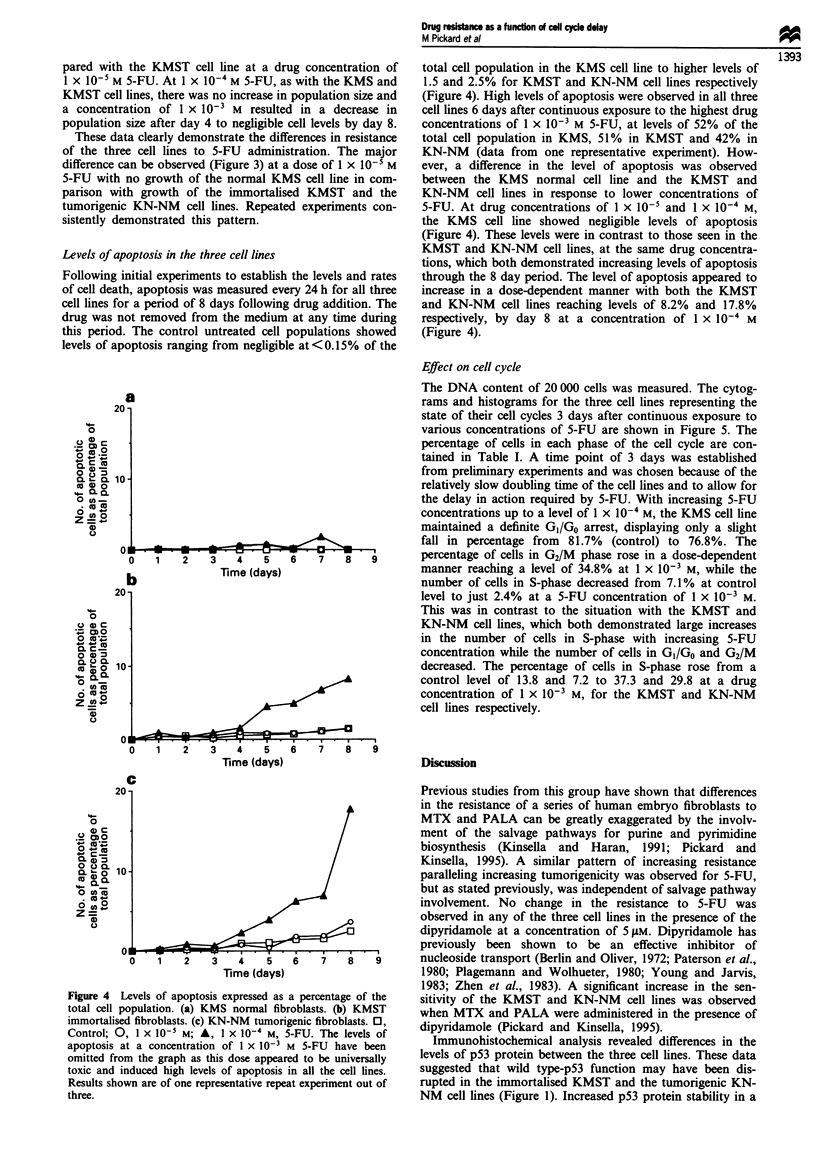

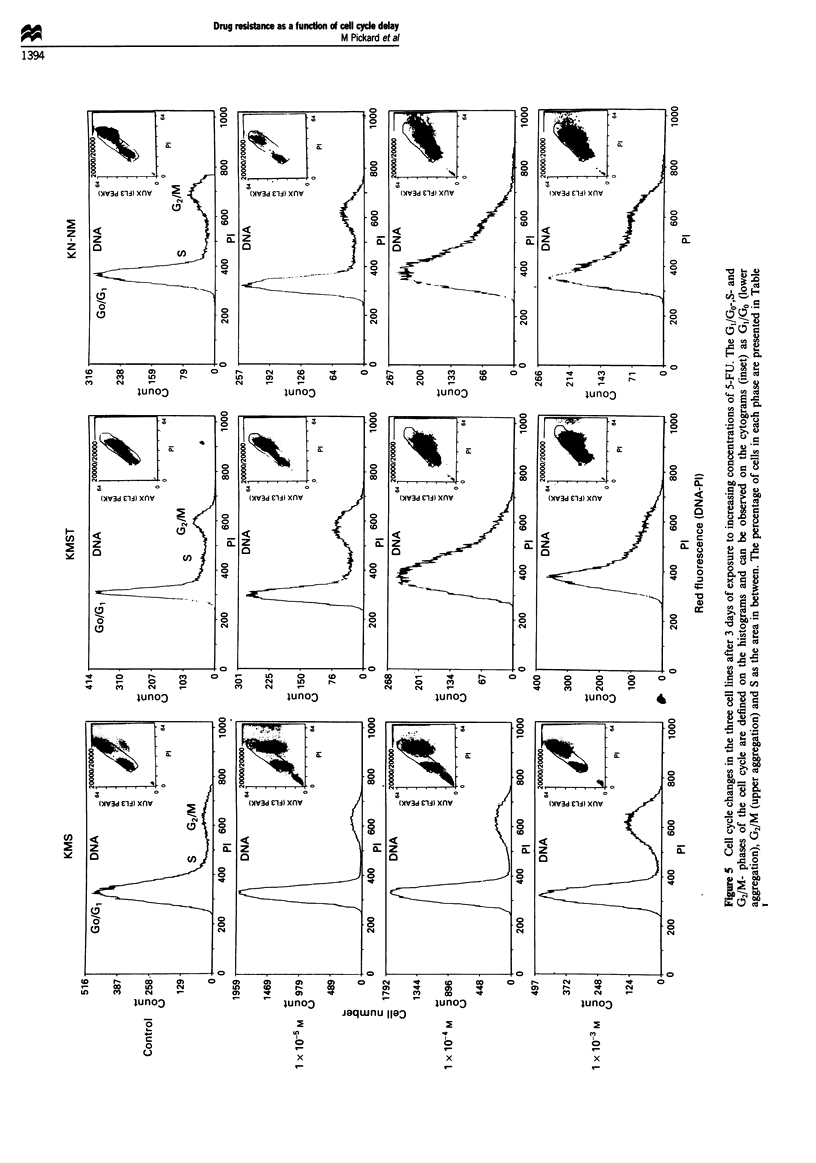

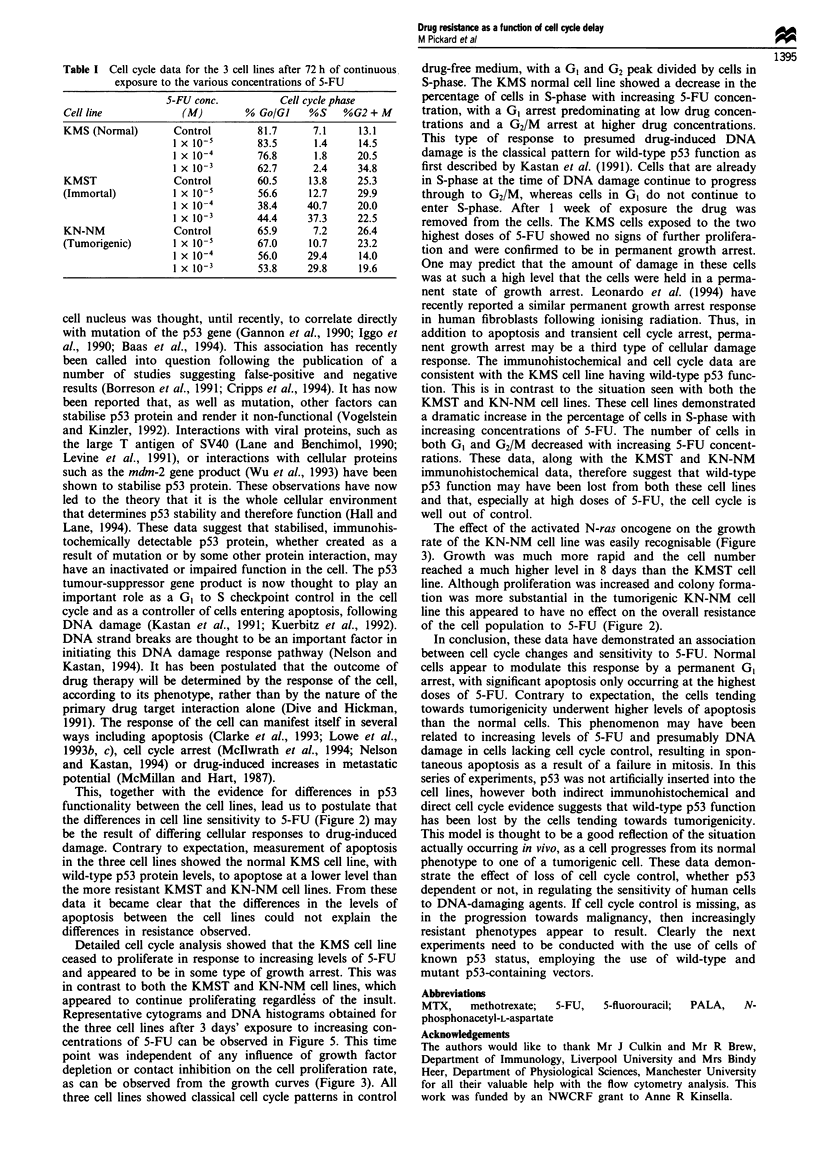

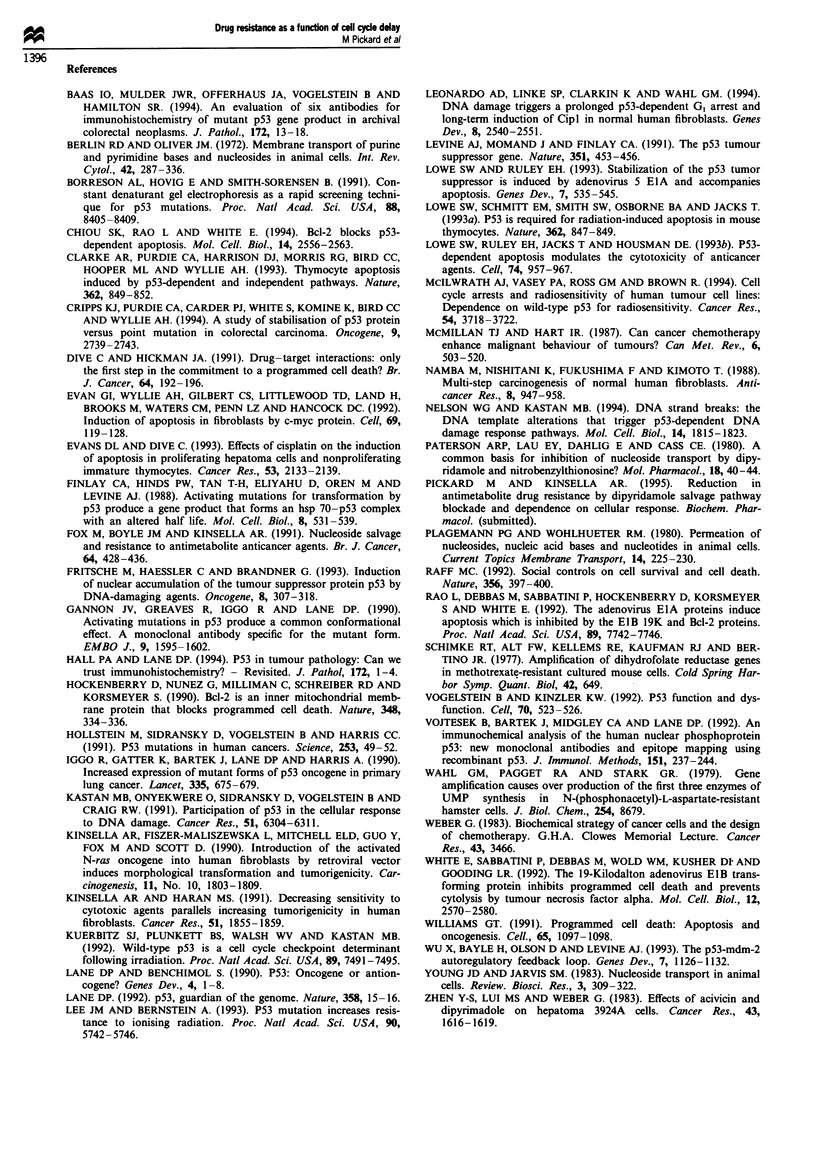


## References

[OCR_01005] Berlin R. D., Oliver J. M. (1975). Membrane transport of purine and pyrimidine bases and nucleosides in animal cells.. Int Rev Cytol.

[OCR_01008] Børresen A. L., Hovig E., Smith-Sørensen B., Malkin D., Lystad S., Andersen T. I., Nesland J. M., Isselbacher K. J., Friend S. H. (1991). Constant denaturant gel electrophoresis as a rapid screening technique for p53 mutations.. Proc Natl Acad Sci U S A.

[OCR_01014] Chiou S. K., Rao L., White E. (1994). Bcl-2 blocks p53-dependent apoptosis.. Mol Cell Biol.

[OCR_01021] Clarke A. R., Purdie C. A., Harrison D. J., Morris R. G., Bird C. C., Hooper M. L., Wyllie A. H. (1993). Thymocyte apoptosis induced by p53-dependent and independent pathways.. Nature.

[OCR_01027] Cripps K. J., Purdie C. A., Carder P. J., White S., Komine K., Bird C. C., Wyllie A. H. (1994). A study of stabilisation of p53 protein versus point mutation in colorectal carcinoma.. Oncogene.

[OCR_01118] Di Leonardo A., Linke S. P., Clarkin K., Wahl G. M. (1994). DNA damage triggers a prolonged p53-dependent G1 arrest and long-term induction of Cip1 in normal human fibroblasts.. Genes Dev.

[OCR_01030] Dive C., Hickman J. A. (1991). Drug-target interactions: only the first step in the commitment to a programmed cell death?. Br J Cancer.

[OCR_01038] Evan G. I., Wyllie A. H., Gilbert C. S., Littlewood T. D., Land H., Brooks M., Waters C. M., Penn L. Z., Hancock D. C. (1992). Induction of apoptosis in fibroblasts by c-myc protein.. Cell.

[OCR_01041] Evans D. L., Dive C. (1993). Effects of cisplatin on the induction of apoptosis in proliferating hepatoma cells and nonproliferating immature thymocytes.. Cancer Res.

[OCR_01046] Finlay C. A., Hinds P. W., Tan T. H., Eliyahu D., Oren M., Levine A. J. (1988). Activating mutations for transformation by p53 produce a gene product that forms an hsc70-p53 complex with an altered half-life.. Mol Cell Biol.

[OCR_01052] Fox M., Boyle J. M., Kinsella A. R. (1991). Nucleoside salvage and resistance to antimetabolite anticancer agents.. Br J Cancer.

[OCR_01057] Fritsche M., Haessler C., Brandner G. (1993). Induction of nuclear accumulation of the tumor-suppressor protein p53 by DNA-damaging agents.. Oncogene.

[OCR_01062] Gannon J. V., Greaves R., Iggo R., Lane D. P. (1990). Activating mutations in p53 produce a common conformational effect. A monoclonal antibody specific for the mutant form.. EMBO J.

[OCR_01073] Hockenbery D., Nuñez G., Milliman C., Schreiber R. D., Korsmeyer S. J. (1990). Bcl-2 is an inner mitochondrial membrane protein that blocks programmed cell death.. Nature.

[OCR_01082] Iggo R., Gatter K., Bartek J., Lane D., Harris A. L. (1990). Increased expression of mutant forms of p53 oncogene in primary lung cancer.. Lancet.

[OCR_01088] Kastan M. B., Onyekwere O., Sidransky D., Vogelstein B., Craig R. W. (1991). Participation of p53 protein in the cellular response to DNA damage.. Cancer Res.

[OCR_01092] Kinsella A. R., Fiszer-Maliszewska L., Mitchell E. L., Guo Y. P., Fox M., Scott D. (1990). Introduction of the activated N-ras oncogene into human fibroblasts by retroviral vector induces morphological transformation and tumorigenicity.. Carcinogenesis.

[OCR_01097] Kinsella A. R., Haran M. S. (1991). Decreasing sensitivity to cytotoxic agents parallels increasing tumorigenicity in human fibroblasts.. Cancer Res.

[OCR_01102] Kuerbitz S. J., Plunkett B. S., Walsh W. V., Kastan M. B. (1992). Wild-type p53 is a cell cycle checkpoint determinant following irradiation.. Proc Natl Acad Sci U S A.

[OCR_01110] Lane D. P. (1992). Cancer. p53, guardian of the genome.. Nature.

[OCR_01113] Lee J. M., Bernstein A. (1993). p53 mutations increase resistance to ionizing radiation.. Proc Natl Acad Sci U S A.

[OCR_01122] Levine A. J., Momand J., Finlay C. A. (1991). The p53 tumour suppressor gene.. Nature.

[OCR_01136] Lowe S. W., Ruley H. E., Jacks T., Housman D. E. (1993). p53-dependent apoptosis modulates the cytotoxicity of anticancer agents.. Cell.

[OCR_01128] Lowe S. W., Ruley H. E. (1993). Stabilization of the p53 tumor suppressor is induced by adenovirus 5 E1A and accompanies apoptosis.. Genes Dev.

[OCR_01131] Lowe S. W., Schmitt E. M., Smith S. W., Osborne B. A., Jacks T. (1993). p53 is required for radiation-induced apoptosis in mouse thymocytes.. Nature.

[OCR_01141] McIlwrath A. J., Vasey P. A., Ross G. M., Brown R. (1994). Cell cycle arrests and radiosensitivity of human tumor cell lines: dependence on wild-type p53 for radiosensitivity.. Cancer Res.

[OCR_01149] McMillan T. J., Hart I. R. (1987). Can cancer chemotherapy enhance the malignant behaviour of tumours?. Cancer Metastasis Rev.

[OCR_01152] Namba M., Nishitani K., Fukushima F., Kimoto T. (1988). Multistep carcinogenesis of normal human fibroblasts. Human fibroblasts immortalized by repeated treatment with Co-60 gamma rays were transformed into tumorigenic cells with Ha-ras oncogenes.. Anticancer Res.

[OCR_01159] Nelson W. G., Kastan M. B. (1994). DNA strand breaks: the DNA template alterations that trigger p53-dependent DNA damage response pathways.. Mol Cell Biol.

[OCR_01164] Paterson A. R., Lau E. Y., Dahlig E., Cass C. E. (1980). A common basis for inhibition of nucleoside transport by dipyridamole and nitrobenzylthioinosine?. Mol Pharmacol.

[OCR_01177] Raff M. C. (1992). Social controls on cell survival and cell death.. Nature.

[OCR_01181] Rao L., Debbas M., Sabbatini P., Hockenbery D., Korsmeyer S., White E. (1992). The adenovirus E1A proteins induce apoptosis, which is inhibited by the E1B 19-kDa and Bcl-2 proteins.. Proc Natl Acad Sci U S A.

[OCR_01187] Schimke R. T., Alt F. W., Kellems R. E., Kaufman R. J., Bertino J. R. (1978). Amplification of dihydrofolate reductase genes in methotrexate-resistant cultured mouse cells.. Cold Spring Harb Symp Quant Biol.

[OCR_01193] Vogelstein B., Kinzler K. W. (1992). p53 function and dysfunction.. Cell.

[OCR_01199] Vojtesek B., Bártek J., Midgley C. A., Lane D. P. (1992). An immunochemical analysis of the human nuclear phosphoprotein p53. New monoclonal antibodies and epitope mapping using recombinant p53.. J Immunol Methods.

[OCR_01205] Wahl G. M., Padgett R. A., Stark G. R. (1979). Gene amplification causes overproduction of the first three enzymes of UMP synthesis in N-(phosphonacetyl)-L-aspartate-resistant hamster cells.. J Biol Chem.

[OCR_01209] Weber G. (1983). Biochemical strategy of cancer cells and the design of chemotherapy: G. H. A. Clowes Memorial Lecture.. Cancer Res.

[OCR_01217] White E., Sabbatini P., Debbas M., Wold W. S., Kusher D. I., Gooding L. R. (1992). The 19-kilodalton adenovirus E1B transforming protein inhibits programmed cell death and prevents cytolysis by tumor necrosis factor alpha.. Mol Cell Biol.

[OCR_01223] Williams G. T. (1991). Programmed cell death: apoptosis and oncogenesis.. Cell.

[OCR_01225] Wu X., Bayle J. H., Olson D., Levine A. J. (1993). The p53-mdm-2 autoregulatory feedback loop.. Genes Dev.

[OCR_01229] Young J. D., Jarvis S. M. (1983). Nucleoside transport in animal cells.. Biosci Rep.

[OCR_01233] Zhen Y. S., Lui M. S., Weber G. (1983). Effects of acivicin and dipyridamole on hepatoma 3924A cells.. Cancer Res.

